# Molecular cloning, characterization and expression analysis of a novel *PDRG1* gene from black tiger shrimp *(Penaeus monodon)*

**DOI:** 10.1590/1678-4685-GMB-2016-0144

**Published:** 2017-03-02

**Authors:** Chao Zhao, Wenting Dai, Lihua Qiu

**Affiliations:** 1 South China Sea Fisheries Research Institute, Chinese Academy of Fishery Sciences, Guangzhou, China; 2 College of Aqua-life Science and Technology, Shanghai Ocean University, Shanghai, China; 3 Key Laboratory of South China Sea Fishery Resources Exploitation & Utilization, Ministry of Agriculture, Guangzhou, China; 4 Tropical Aquaculture Research and Development Center of South China Sea Fisheries Research Institute, Sanya, China

**Keywords:** PDRG1, gene cloning, RT-qPCR, ovarian development, black tiger shrimp

## Abstract

*P53 And DNA Damage-Regulated Gene 1 (PDRG1)* is a novel gene which plays an important role in chaperone-mediated protein folding. In the present study, the full-length complementary DNA (cDNA) sequence of the *PDRG1* gene from *Penaeus monodon* (*PmPDRG1*) was cloned by the rapid amplification of cDNA ends (RACE) method. The cDNA of *PmPDRG1* spans 1,613 bp, interrupted by only one short intron, and encodes a protein of 136 amino acids with calculated molecular weight of 15.49 kDa. The temporal expression profile of *PmPDRG1* in different tissues and in different developmental stages of the ovary was investigated by real-time quantitative PCR (RT-qPCR). An RNA interference (RNAi) experiment was performed to study the relationship between *P. monodon p53* (*Pmp53*) and *PmPDRG1*, and the results showed that the relative expression level of *PmPDRG1* mRNA was notably up-regulated from 12 h to 96 h after *Pmp53* was silenced both in ovary and hepatopancreas. To further explore the role of PmPDRG1 in ovarian development, dopamine (DA) and 5-hydroxytryptamine (5-HT)-injected shrimps were analyzed by RT-qPCR, indicating that PmPDRG1 may be involved in the regulation of ovarian development of *P. monodon*.

## Introduction

*P53 And DNA Damage-Regulated Gene 1* (*PDRG1*, namely was first identified in 2003 by Luo *et al*. (2003). The human *PDRG1* resides at the long arm of chromosome 20 and encodes a protein of 133 amino acids that is present within a distinct subcellular compartment of the cytoplasm (Deloukas *et al*., 2001; Luo *et al*., 2003). *PDRG1* is usually strongly over-expressed in multiple human malignancies (Jiang *et al*., 2011; Wang *et al*., 2015a). PDRG1 protein was identified as a subunit of the R2TP/prefoldin-like complex, which is involved in the assembly of the RNA polymerase II complex (Pol II) in the cytoplasm of eukaryotic cells (Sardiu *et al*., 2008; Boulon *et al*., 2012; Mita *et al*., 2013). The tumor suppressor protein p53 can down-regulate the expression of *PDRG1* mRNA, while ultraviolet (UV) radiation has the opposite effect (Luo *et al*., 2003). As certain interactions between PDRG1 and p53 exist, some scholars believe that PDRG1 has the potential to be a novel valuable tumor biomarker that could play a role in cancer development and/or progression or to be a DNA damage-associated maker (Jiang *et al*., 2011; Saigusa *et al*., 2012; Wang *et al*., 2015a). Furthermore, PDRG1 is proven to be involved in apoptosis and cell cycle regulation (Jiang *et al*., 2011; Wang *et al*., 2014). Although the roles of PDRG1 in DNA damage and tumor cell growth in vertebrates have been widely studied, the functions of PDRG1 in invertebrates, especially in crustaceans, are poorly understood.

The black tiger shrimp (*P. monodon*) is one of the most important aquatic commercial animals in Asia, especially in southern China. Because the eyestalk of *P. monodon* can secrete ovarian suppression hormones, unilateral eyestalk ablation is usually adopted to induce ovarian maturation of *P. monodon*, but this technique leads to the death of parent shrimps and lowers spawning quality (Benzie, 1998; Phinyo *et al*., 2013). Therefore, it is imperative to explore alternative technologies to eyestalk ablation and to understand the molecular mechanisms that control the development and maturation of ovaries/oocytes (Hiransuchalert *et al*., 2013; Phinyo *et al*., 2014). In our previous study, we found that Pmp53 plays an important role in the development and maturation of the ovaries in *P. monodon* (Dai *et al*., 2016). In the present study, we silenced *Pmp53* to investigate its relationship with *PmPDRG1* and the role that *PmPDRG1* may play in the ovarian development of *P. monodon*. Biogenic amines such as dopamine (DA) and serotonin (5-hydroxytryptamine, 5-HT) are able to affect numerous physiological processes in crustaceans through their actions as neuroregulators. Both DA and 5-HT have been shown to be involved in the synthesis and release of neurohormones, such as crustacean hyperglycemic hormone (CHH), vitellogenesis-inhibiting hormone (VIH) and molt-inhibiting hormone (MIH) (Chen *et al*., 2003). It has been demonstrated that injected 5-HT can induce ovarian maturation in shrimp (Vaca and Alfaro, 2000), while dopamine depressed vitellogenin synthesis (Chen *et al*., 2003). The relationship between PDRG1 and ovarian maturation and the effects of DA and 5-HT on the expression levels of *PmPDRG1* in ovaries and hepatopancreas of *P. monodon* are presented in this paper.

In this study, we cloned and characterized the full-length cDNA of *P. monodon* *PmPDRG1* and assessed the distribution of *PmPDRG1* transcripts in different tissues and ovary developmental stages. In addition, we investigated the expression profiles of *PmPDRG1* mRNA in selected tissues after injection of Pmp53-dsRNA and exposure to 5-HT and DA. Results from this study will contribute to a better understanding of *PDRG1* and its function in ovarian development of *P. monodon*.

## Materials and Methods

### Experimental animals and sample collection

Healthy black tiger shrimp, *P. monodon* (100 ± 18 g), cultivated in aerated seawater (salinity of 30 PSU) for three days at 25 ± 1 °C in the Shenzhen Base of South China Sea Fisheries Research Institute (Shenzhen, Guangdong province, China) were used as the material in the experiment. Various tissues (ovary, heart, intestine, brain, muscles, stomach and gills) from male and female individuals were dissected, snap frozen in liquid nitrogen, and stored at −80 °C. Five shrimp in each ovarian maturation stages were selected. The different ovarian stages used in this study were classified according to the morphology reported by Huang *et al*. (2006), as ovogonium stage (I), chromatin nucleolus stage (II), perinucleolus stage (III), yolky stage (IV), and cortical rod stage (V).

### Total RNA extraction, first strand cDNA synthesis and DNA extraction

Total RNA was isolated from the examined tissue (about 100 mg) of the shrimp using TRIzol (Invitrogen, Shanghai, China) reagent following the manufacturer' protocol, resuspended in DEPC-treated water and stored at –80 °C (Jiang *et al*., 2009). The concentration of RNA was determined using a NanoDrop 2000 spectrometer (Thermo, USA), and RNA integrity was assessed by 1% agarose gel electrophoresis. The cDNA was synthesized from 1 μg of mRNA using a PrimeScript Reverse Transcriptase kit (TaKaRa, Dalian, China) following the manufacturer's protocol, as previously described (Wang *et al*., 2015b). The cDNA was used as the template for PCR reactions in gene cloning. The phenol-chloroform-isoamyl alcohol method was used to isolate total genomic DNA, which was then used as template to amplify introns.

### Gene cloning and sequencing

A partial sequence of *PDRG1* (970 bp) was isolated from the transcriptome database. Initially, PCR was carried out using the cDNA described above as template, using the primers PaF and PaR (Table 1) designed according to the partial sequence of *PDRG1*, for verification. Then, the 3′ end cDNA sequence of the *PDRG1* gene was isolated using a SMART^TM^ RACE cDNA amplification kit (Clontech, Takara) (Jiang *et al*., 2009; Wang *et al*., 2015b). In the 3′RACE PCR, the touchdown PCR step was performed with the gene-specific primer pdrg-sp1 and a universal primer UPM (a mix of UPX-long and UPX-short, Table 1). The PCR cycling parameters were as follows: an initial denaturation at 94 °C for 3 min, followed by 30 cycles at 94 °C for 30 s, 60 °C for 30 s, and 72 °C for 3 min, and the last cycle was followed by 10 min extension at 72 °C. Additionally, a nested PCR with pdrg-sp2 and NUP was carried out (PCR profile was as follows: 94 °C for 3 min; 94 °C for 30 s, 55 °C for 30 s, 72 °C for 3 min in 35 cycles; 72 °C for 10 min). The PCR products were purified using a PCR purification kit (Sangon, Shanghai, China) and cloned into the pMD18-T vector (TaKaRa). After transformtion into competent cells (*E.coli* DH5α), the positive clones were sequenced in both directions (Invitrogen, Guangzhou, China), and the resulting sequences were verified and subjected to cluster analysis.

**Table 1:** Primers used for gene cloning and expression analysis.

Name	Primer sequence (5′ → 3′)	Application
3′-CDS	AAGCAGTGGTATCAACGCAGAGTAC(T)30VN	reverse transcription
PaF	CTGGTGATGCAAGTGCAGTTCAG	PCR
PaR	TCCTCCTTACAATGAACTGTGCCA	PCR
pdrg-sp1	GGAACGAACCTCAAGCCCTTATCACAA	3′RACE
pdrg-sp2	AAAATGTAGGGGGAGAAACTGTAGAAGC	3′RACE
UPX-long	CTAATACGACTCACTATAGGGCAAGCAGTGGTATCAACGCAGAGT	3′RACE
UPX-short	CTAATACGACTCACTATAGGGC	3′RACE
NUP	AAGCAGTGGTATCAACGCAGAGT	3′RACE
qpdrg -F	TGCGGCAGAGGATGTTATATCA	Real time PCR
qpdrg-R	CCTGTGGACTGACTGGCTAAT	Real time PCR
rEF-F	AAGCCAGGTATGGTTGTCAACTTT	Real time PCR
rEF-R	CGTGGTGCATCTCCACAGACT	Real time PCR
pdrg B F	CGCGGATCCATGGCAGTGTCTCCAGAACGTATC	Prokaryotic expression
pdrg B R	CCCAAGCTTTCTACCAAGAACTTGCTTTACAGCT	Prokaryotic expression
pdrg NF1	TGCGGCAGAGGATGTTATATCA	Intron amplification
pdrg NR1	CCCAAGCTTTCTACCAAGAACTTGCTTTACAGCT	Intron amplification
pdrg NF2	CGCGGATCCATGGCAGTGTCTCCAGAACGTATC	Intron amplification
pdrg NR2	CCCAAGCTTTCTACCAAGAACTTGCTTTACAGCT	Intron amplification

### Sequence analysis, multiple sequence alignment, and phylogenetic analysis

The obtained *PmPDRG1* cDNA sequence was compared with other known sequences in the NCBI database using the Blast algorithm (Altschul *et al*., 1997). The open reading frame (ORF) of *PmPDRG1* cDNA was determined using ORF Finder software (http://www.ncbi.nlm.nih.gov/projects/gorf/). The molecular weight and pI of the deduced PmPDRG1 protein were examined using the Compute pI/Mw tool of the Expasy server (http://web.expasy.org/compute_pi/). N-glycosylation site prediction was done using NetNGlyc 1.0 software (http://www.cbs.dtu.dk/services/NetNGlyc/) and phosphorylation sites were predicted using NetPhos2.0 (http://www.cbs.dtu.dk/services/NetPhos/). Multiple sequence alignment was performed using the ClustalX 2.0.11 software. The signal peptide was predicted using the Signal P 4.1 program (http://www.cbs.dtu.dk/services/SignalP/) and the SMART 4.0 program (http://smart.embl-heidelberg.de/) was used to predict functional sites or domains in the amino acid sequence. A phylogenetic tree was constructed by the neighbor-joining (NJ) method and support of a bootstrap analysis with 1,000 replications implemented in the MEGA 5.0 package (Chen *et al*., 2013).

### RNA interference

To investigate the relationship between PmPDRG1 and Pmp53 in ovarian development, an RNAi experiment was carried out using dsRNA specific for *Pmp53*. An *in vitro* expression system was adopted to obtain dsRNA. In brief, the recombined pD7 vector containing a bidirectional T7 RNA polymerase promoter was constructed using the pUC18 vector as described in previous studies (Robalino *et al*., 2007; Wang *et al*., 2010).

Subsequently, a recombinant plasmid (pD7-p53) containing a reverse complement of *p53* was established and used as PCR template. Two separate PCR assays were set up with the primers pF/i53-R and pR/i53-F (Table 1). The PCR products of 763 bp and 590 bp were excised, gel-purified and used for *in vitro* transcription. Subsequently, dsRNA-p53 was synthesized using the *in vitro* Transcription T7 kit (TaKaRa) according to the manufacturer's instructions. The quality of dsRNAs was verified by 1.5% agarose gel electrophoresis and quantified using UV spectrophotometry. The dsRNA was stored at −80 °C until the experiment.

*P. monodon* shrimps (100 ± 2 g, 14 months old) were acclimatized for 2 days before dsRNA-p53 injection. The shrimps were injected with 300 μg of dsRNA-p53 dissolved in 40 μL of sterilized saline solution (10 mM Tris-HCl pH 7.5, 400 mM NaCl). Shrimps injected with sterilized saline solution were used as vehicle control (VC). Ovaries and hepatopancreas of the shrimp were randomly collected at 0, 12, 24, 48, 72, 96 h after injection, and frozen in liquid nitrogen for future RT-qPCR analysis.

### 5-HT and dopamine challenge

To examine the effects of 5-HT and DA on the expression levels of *PmPDRG1*, seven groups of female shrimp (100 ± 18 g, 14 months old) were injected intramuscularly into the first abdominal segment with 5-HT and DA (50 μg/g body weight). Shrimp injected with sterilized saline solution at 0 h were included as control. The ovaries and hepatopancreas were collected at 0, 6, 12, 24, 48, 72, 96 h post injection, and preserved in liquid nitrogen for future RT-qPCR analysis.

### RT-qPCR for gene expression profile analysis

RT-qPCR was used to detect the temporal expression of the genes. cDNA was synthesized using the PrimeScript^TM^ RT reagent Kit with gDNA Eraser (Perfect Real Time) (TaKaRa) and used as templates for RT-qPCR assays. RT-qPCR was performed using SYBR^®^ *Premix Ex Taq* ^TM^ II(TaKaRa) as described in our previous study (Jiang *et al*., 2009). The reference gene *elongation factor-1alpha* (*EF-1*α) (GenBank: DQ021452.1) was used as internal control for normalizing the cDNA template by menas of the primers rEF-F and rEF-R (Table 1). Each 25 μL reaction solution contained: 12.5 μL of 2SYBR^®^ *Premix Ex Taq* II, 0.5 μL of forward primer (10 μM), 0.5 μL of reverse primer (10 μM), 2 μL of cDNA template equivalent to 70 ng total RNA, and 9.5 μL sterile distilled water. Each reaction was carried out simultaneously in three separate tubes and the test was repeated three times (Li *et al*., 2013). Thermal cycling conditions were 95 °C for 30 s, followed by 42 cycles of 95 °C for 5 s, 60 °C for 30 s. A melting curve analysis was added (95 °C for 1 s, 65 °C for 15 s, 95 °C for continuous acquisition) to demonstrate the specificity of the PCR products, as revealed by a single peak. The 2^−ΔΔCT^ method was used to calculate relative gene expression levels (Livak and Schmittgen, 2001; Qiu *et al*., 2010).

### Statistical analysis

Statistical analyses were carried out using SPSS software (SPSS Inc, USA). Data are reported as mean ± standard error (SE). Results obtained from qRT-PCR analysis were subjected to one-way analysis of variance (one-way ANOVA) followed by an unpaired, two-tailed *t*-test. Differences were considered significant at P < 0.05.

## Results

### Characterization of the *PmPDRG1* full-length cDNA

**Figure 1:**
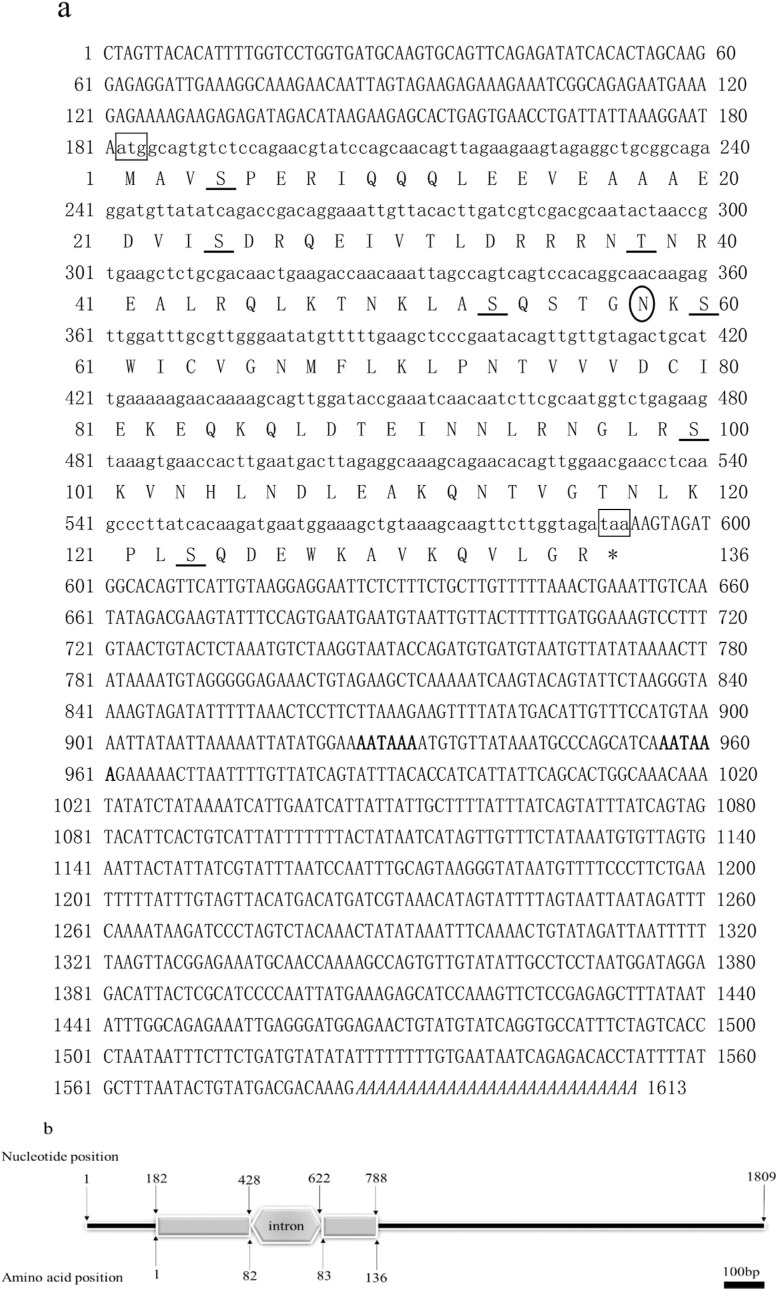
Deduced amino acid and sequences of *PmPDRG1*. (a) Numbers on the right and left of each row represent amino acid or nucleotide position; sequences in the boxes represent the start codon (ATG) and termination codon (TAA); eukaryotic polyadenylation signals AATAAA are highlighted in bold; the polyA signal sequence is italicized; the N-glycosylation site is marked by a circle; phosphorylation sites are underlined. (b) Schematic diagram representing the genomic DNA region of *PmPDRG1*.

The full-length cDNA sequence of *PmPDRG1* was obtained by RACE-PCR, and the complete nucleotide sequence and the deduced amino acid sequence are shown in Figure 1a (GenBank: KX156929). The cDNA sequence of *PmPDRG1* is 1,613 bp in length, including an open reading frame of 411 bp (position 182-592 bp), a 5′UTR of 181 bp and a 3′UTR of 1,021 bp. Two eukaryotic polyadenylation signals AATAAA were located between nucleotides 926-931 bp and 956-961 bp and a poly (A) tail 28 bp downstream. The ORF sequence was predicted to encode a protein of 136 amino acids with a calculated molecular mass of about 15.49 kDa, and a theoretical isoelectric point of 8.62.

The structure prediction results showed that PmPDRG1 contains 66.17% of α-helix, 5.15% of β-pleated sheet, and 28.68% of random coil. These conserved regions include seven phosphorylation sites and one N-glycosylation site (Figure 1a). Signal P 4.1 analysis revealed that PmPDRG1 does not contain a typical signal peptide sequence. *PmPDRG1* is encoded in the nuclear genome, and has only one intron in the ORF (Figure 1b).

### Phylogenetic analysis of PmPDRG1

**Figure 2:**
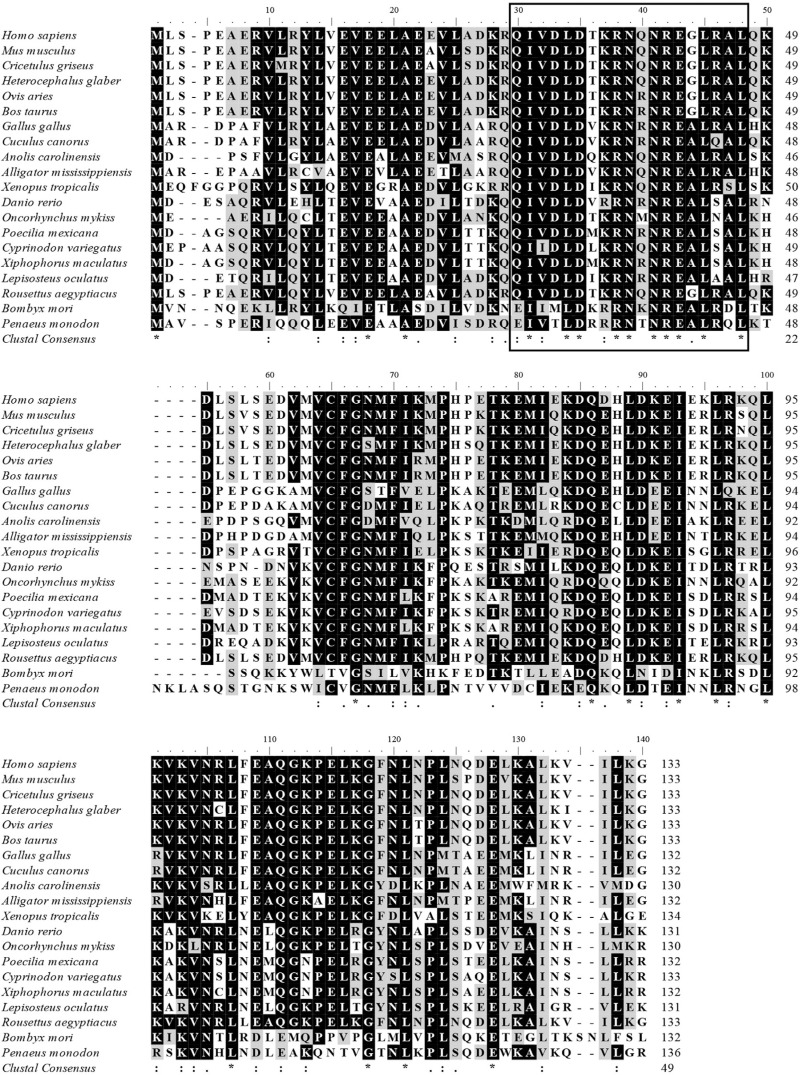
Multiple alignments of the deduced amino acid sequence of *PmPDRG1* with other known *PDRG1* aligned by Clustal X 2. 0. 11. Identical and similar sites are indicated with asterisks (*) and dots (. or :) , respectively. Black rectangles represent the phylogenetically conserved domain in different species. The species names and GenBank accession numbers are as follows: *Homo sapiens* (NP_110442.1); *Mus musculus* (NP_849270.1); *Cricetulus griseus* (ERE71423.1); *Heterocephalus glaber* (EHB11715.1); *Ovis aries* (XP_004014522.1); *Bos taurus* (NP_001071583.1); *Gallus gallus* (XP_015151961.1); *Cuculus canorus* (XP_009560934.1); *Anolis carolinensis* (XP_008119059.1); *Alligator mississippiensis* (KYO24169.1); *Xenopus tropicalis* (NP_001015688.1); *Danio rerio* (NP_001017757.1); *Oncorhynchus mykiss* (NP_001154150.1); *Poecilia mexicana* (XP_014829745.1); *Cyprinodon variegatus* (XP_015254726.1); *Xiphophorus maculatus* (XP_005810586.1); *Lepisosteus oculatus* (XP_006639564.1); *Rousettus aegyptiacus* (XP_016004320.1); *Bombyx mori* (XP_004928502.1); *Penaeus monodon* (KX156929).

The predicted amino acid sequence shared homology with previously published PDRGR1 sequences of other species in the GenBank database, detected using BLAST program. These include a 49% identity with *Anoplopoma fimbria* (ACQ58385.1), 48% identity with *Oncorhynchus mykiss* (NP_001154150.1), and 47% identity with *Danio rerio* (NP_001017757.1). The putative amino acid sequence of PmPDRG1 was aligned with other species in conserved regions. The deduced amino acid sequence QIVDLDTKRNQNREALRAL (30-48aa) of PmPDRG1 shared high homology with other species (Figure 2).

**Figure 3:**
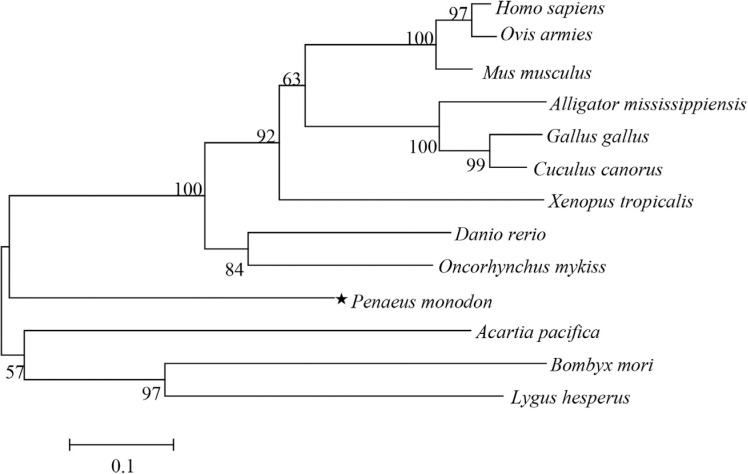
NJ phylogenetic tree based on amino acid sequence encoded by PDRG1 as revealed by MEGA 5. 0 software. The numbers above the branches represent bootstrap values (1000 replicates). The following PDRG1 proteins family members were used in the phylogenetic analysis: *Homo sapiens* (NP_110442.1); *Ovis aries* (XP_004014522.1); *Mus musculus* (NP_849270.1); *Alligator mississippiensis* (KYO24169.1); *Gallus gallus* (XP_015151961.1); *Cuculus canorus* (XP_009560934.1); *Xenopus tropicalis* (NP_001015688.1); *Danio rerio* (NP_001017757.1); *Oncorhynchus mykiss* (NP_001154150.1); *Penaeus monodon* (KX156929); *Acartia pacifica* (ALS04393.1); *Bombyx mori* (XP_004928502.1); *Lygus Hesperus* (JAG33482.1).

As shown in Figure 3, the PDRG1 phylogenetic tree comprised two main clusters: the upper cluster contained vertebrate PDRG1 sequences, while the second cluster contained invertebrate PDRG1 sequences. Vertebrate PDRG1 proteins appeared closely related to each other and converged into one subgroup, which include PmPDRG1. Although the shrimp PmPDRG1 was more similar to the vertebrate subgroup than the other main branch that includes the majority of the invertebrates, it still was an outlier in the main branch of the vertebrate subgroup.

### Tissue expression analysis of *PmPDRG1*

**Figure 4:**
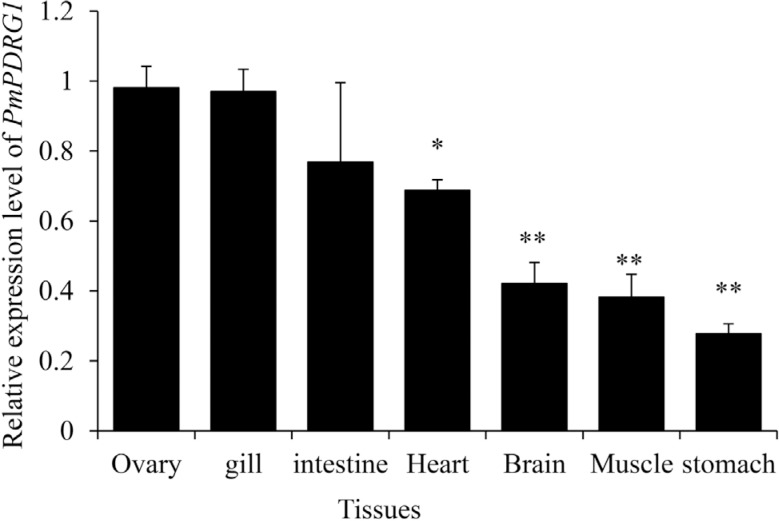
Relative expression levels of *PmPDRG1* mRNA in tissues. Tissue distribution of *PDRG1* transcripts in the shrimp by RT-qPCR analysis using *EF-1*α as an internal reference. Vertical bars represented mean ± SD (n = 5). Significant differences between the experimental and the control group are indicated by asterisks * (*P* < 0.05); ** (*P* < 0.01).

The tissue distribution pattern of *PmPDRG1* mRNA is shown in Figure 4. The RT-qPCR results proved that the *PmPDRG1* gene was expressed in all the examined tissues, with relatively high levels in the ovary, gill and intestine, moderate levels in the heart and brain, and low levels in th muscle and stomach (Figure 4).

### Expression profiles of *PmPDRG1* mRNA during ovarian maturation stages

**Figure 5:**
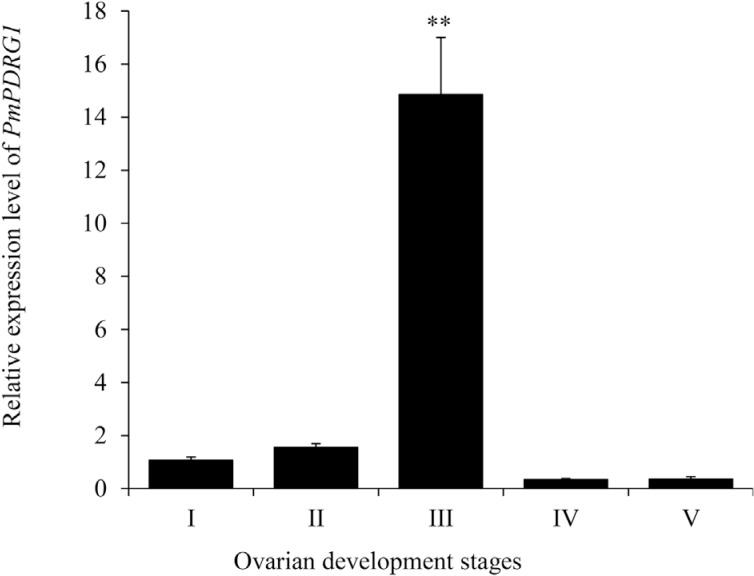
Relative expression levels of *PmPDRG1* mRNA at different developmental stages of the ovaries. I, ovogonium stage; II, chromatin nucleolus stage; III, perinucleolus stage; IV, yolky stage; and V, cortical rod stage; Vertical bars represent the mean ± SD (n =5). ** *P* < 0.01.

The relative expression levels of *PmPDRG1* mRNA in different ovarian stages of *P. monodon* were investigated by RT-qPCR. The expression level in stage III ovaries was about 14.5-fold higher than in other stages (*P* < 0.05) as shown in Figure 5. The expression among stages I, II, IV, and V were not significantly different.

### *PmPDRG1* mRNA expression profiles after *Pmp53* gene silencing by Pmp53-dsRNA

**Figure 6:**
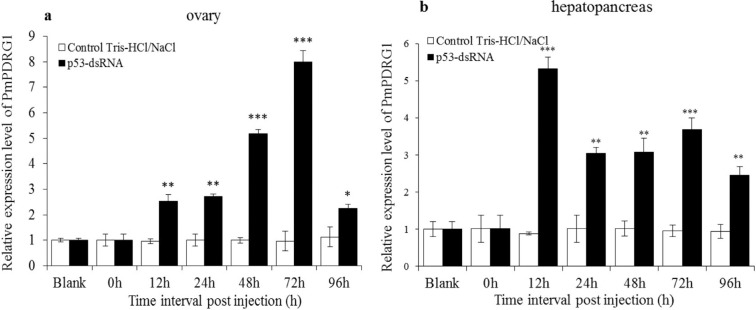
*PmPDRG1* mRNA expression profiles after silencing by Pmp53-dsRNA. (a) *PmPDRG1* relative expression levels in ovary tissue post treatment with Pmp53-dsRNA; (b) *PmPDRG1* relative expression levels in hepatopancreas tissue post treatment with Pmp53-dsRNA. Vertical bars represent the mean ± SD (n =3). Significant differences between the experimental and the control group are indicated by asterisks. * *P* < 0.05; ** *P* < 0.01; *** *P* < 0.001).

To investigate the relationship between Pmp53 and PmPDRG1, the *Pmp53* gene was silenced by Pmp53-dsRNA. In ovary, the silencing efficiency of *Pmp53* at 12, 24, 48 and 72 h of dsRNA-p53 post-injection were 65.86, 85.35, 64.06 and 25.45%, respectively, and in hepatopancreas, the silencing efficiency from 12, 24, 48, 72 and 96 h post injection were 37.70, 39.29, 64.24, 88.67 and 20.37%, respectively. Detailed data on *Pmp53* gene silencing have been published in our previous study (Dai *et al*., 2016). After *Pmp53* was successfully silenced, the *PmPDRG1* mRNA expression pattern was analyzed by RT-qPCR assays in ovary and hepatopancreas. In ovary, the relative expression levels of *PmPDRG1* mRNA were notably up-regulated at 12, 24, 48, 72 and 96 h post-injection of Pmp53-dsRNA compared to the control group. Additionally, its transcripts showed the lowest level at 96 h and the highest level at 72h post-injection (Figure 6a). In hepatopancreas of *P. monodon*, the relative expression levels of *PmPDRG1* mRNA were notably up-regulated from 12 to 96 h post-injection of Pmp53-dsRNA compared to the control group (Figure 6b). The relative expression levels of *PmPDRG1* mRNA were 5.3, 3.0, 3.1, 3.7 and 2.5-fold higher compared to the control group at 12, 24, 48, 72 and 96 h respectively.

### *PmPDRG1* mRNA expression profiles after stimulation by 5-HT and DA

**Figure 7:**
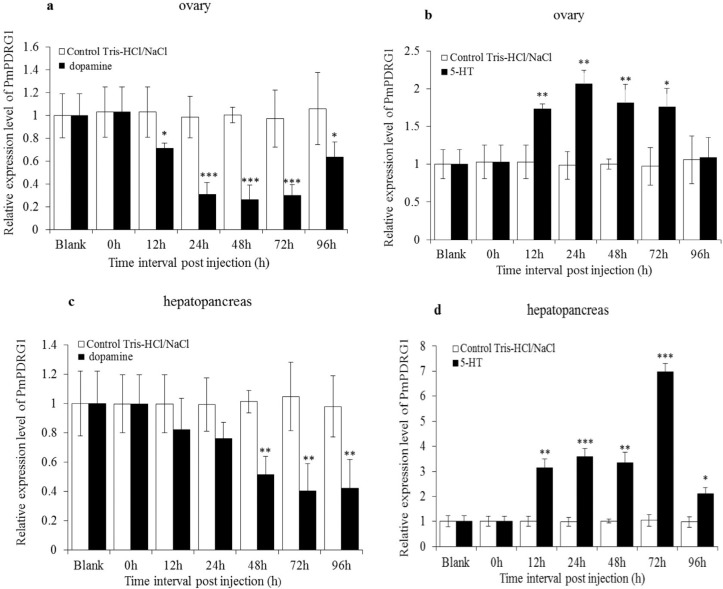
*PmPDRG1* mRNA expression profiles after stimulation with 5-HT and DA. (a) *PmPDRG1* relative expression levels in ovary tissue post treatment with DA. (b) *PmPDRG1* relative expression levels in ovary tissue post treatment with 5-HT. (c) *PmPDRG1* relative expression level in hepatopancreas tissue post treatment with DA. (d) *PmPDRG1* relative expression levels in hepatopancreas tissue post treatment with 5-HT. Vertical bars represent the mean ± SD (n =3). Significant differences between the experimental and the control group are indicated by asterisks * *P* < 0.05; ** *P* < 0.01; *** *P* < 0.001).

The expression levels of *PmPDRG1* at 12–96 h post injection of Tris-HCl/NaCl were not significantly different from the untreated group. After the shrimp were injected with DA, the expression levels of *PmPDRG1* were significantly reduced from 12–96 h in the ovary of *P. monodon* (Figure 7a) and at 48, 72 and 96 h in hepatopancreas of *P. monodon* (Figure 7c). In ovary, the expression levels of *PmPDRG1* were significantly increased after injection of 5-HT at 12, 24, 48 and 72 h compared to the control group (Figure 7b), and in hepatopancreas, at 12–96h post injection. Specifically, after injection of 5-HT the expression level of *PmPDRG1* was 6.98-fold higher compared with the control group at 72h (Figure 7d).

## Discussion

In the present study, the full-length cDNA sequence of the *P. monodon* *PmPDRG1* gene was identified and characterized (Figure 1). Two potential polyadenylation signal sequences (AATAAA) were found in the 3′UTR of *PmPDRG1*, however, only one is found in *PDRG1* of *Apostichopus japonicus* (Wang and Yang, 2013), and human (Luo *et al*., 2003). Although the deduced amino acid sequence QIVDLDTKRNQNREALRAL (30-48aa) of PmPDRG1 shares high homology with other species (Figure 2) (Wang and Yang, 2013), the total protein sequence of PmPDRG1 consists of 136 amino acids, compared to 133 amino acids deduced for PDRG1 of both human and mouse (Luo *et al*., 2003; Wang and Yang, 2013). Luo *et al*. (2003) reported that a helix-turn-helix motif (LNQDELKALKVILKG) exists at the C-terminal end of both human and mouse PDRG proteins, which is involved in protein-protein and protein-DNA interactions. However, we could not find such a motif in PmPDRG1, and further research is needed to study this difference. So far, the function of PDRG1 is still unclear because research about animal *PDRG1* genes is relative rare and lacks thoroughness.

To study the evolutionary relationships of PmPDRG1 with other invertebrate and vertebrate PDRG1 family members, a phylogenetic analysis of the PDRG1 was performed. Vertebrate PDRG1 proteins are closely related to each other and converge into one subgroup, and even though PmPDRG1 was included in this vertebrate subgroup, the relationship was not very obvious. The results of the Blast and phylogenetic analysis suggested that PmPDRG1 iss a new member of the PDRG1 family. But the reason why PmPDRG1 was included in the vertebrate subgroup still needs further study.

The expression pattern in different tissues can indicate to some extent the main function(s) of the respective target gene. The results showed that *PmPDRG1* is widely expressed in all the examined tissues, but especially high relative expression levels were detected in the ovary, gill and intestine (Figure 4). The results further indicate that the *PmPDRG1* gene may play diverse roles in *P. monodon*, and that its main function sites may be the ovary, gill and intestine. The results for *PmPDRG1* expression patterns during different maturation stages of the ovaries showed that *PmPDRG1* mRNA increases sharply in stage III, and this is similar to previous reports in which the peak expression levels of *PmCyclin A* and *PmCDK2*, involved in ovarian development, were found at stage III (Visudtiphole *et al*., 2009; Dai *et al*., 2015). As stage III of ovary development is marked by massive cell proliferation and the presence of oocytes that have accumulated yolk substances in the cytoplasm (Huang *et al*., 2006), the results indicate that PmPDRG1 may be related to the oogenesis stage of ovarian development. In our previous study, we found that Pmp53 plays an important role in the development and maturation of the ovaries in *P. monodon* (Dai *et al*., 2016). To now study the relationship between *Pmp53* and *PmPDRG1* we successfully silenced the *Pmp53* gene (Dai *et al*., 2016) causing an up-regulation of the relative expression of *PmPDRG1* both in the ovary and hepatopancreas, indicating that Pmp53 could down-regulate *PmPDRG1* transcript levels. The molecular regulatory mechanisms, however, still need to be further studied.

The study of molecular regulatory mechanisms related to promotion of reproductive development and maturation have begun to receive more attention,, especially in shrimp reproduction. Oocyte development includes a series of complex cellular events, in which differential genes express in a temporal and spatial fashion to guarantee the proper development of the oocytes or to store transcripts and proteins as maternal factors for early embryogenesis (Qiu *et al*., 2005). Vitellogenin (Vg) is synthesized in both the ovary and the hepatopancreas of *P. monodon* (Urtgam *et al*., 2015), and is a nutritive resource, playing an important role in embryonic growth and gonadal development (Bai *et al*., 2015). That is the reason why we selected ovary and hepatopancreas to perform the DA and 5-HT challenge assay. Molecular effects of DA and 5-HT on the relative expression levels of the *PmpPDRG1* in ovaries and hepatopancreas are first reported in this study. The expression levels of *PmPDRG1* mRNA were reduced after injection of DA, and increased after injection of 5-HT both in ovaries and hepatopancreas. Previous studies have shown that DA depresses vitellogenin synthesis and inhibits ovarian maturation. The expression level change of *PmPDRG1* mRNA after DA or 5-HT injection may imply that *PmPDRG1* is implicated in the regulation of ovarian maturation of *P. monodon*. However, knowledge on the detailed functional mechanisms of PmPDRG1 in ovarian maturation are still limited and require further research.

In summary, the complete cDNA sequence of *PmPDRG1* was isolated and characterized in *P. monodon*. Subsequently, the mRNA distribution pattern of *PmPDRG1* in different tissues and ovarian stages was studied to explore its role in the development and maturation of the ovaries. In addition, the expression pattern of *PmPDRG1* post Pmp53-dsRNA was studied to explore the possible relationship between *Pmp53* and *PmPDRG1*. Molecular effects of 5-HT and DA on the expression regulation of *PmPDRG1* in ovaries and hepatopancreas are first reported in this study, which should help to improve our understanding of the molecular mechanisms of ovarian development in shrimp.
